# Chemotherapy and terminal skeletal muscle differentiation in *WT1‐*mutant Wilms tumors

**DOI:** 10.1002/cam4.1379

**Published:** 2018-03-15

**Authors:** Brigitte Royer‐Pokora, Manfred Beier, Artur Brandt, Constanze Duhme, Maike Busch, Carmen de Torres, Hans‐Dieter Royer, Jaume Mora

**Affiliations:** ^1^ Institute of Human Genetics Heinrich‐Heine University Düsseldorf D‐40225 Germany; ^2^ Department of Oncology Hospital Sant Joan de Deu Barcelona 08950 Spain

**Keywords:** Chemotherapy effect, differentiation response, WT1‐mutant Wilms tumor

## Abstract

Wilms tumors (WT) with *WT1* mutations do not respond well to preoperative chemotherapy by volume reduction, suggesting resistance to chemotherapy. The histologic pattern of this tumor subtype indicates an intrinsic mesenchymal differentiation potential. Currently, it is unknown whether cytotoxic treatments can induce a terminal differentiation state as a direct comparison of untreated and chemotherapy‐treated tumor samples has not been reported so far. We conducted gene expression profiling of 11 chemotherapy and seven untreated *WT1*‐mutant Wilms tumors and analyzed up‐ and down‐regulated genes with bioinformatic methods. Cell culture experiments were performed from primary Wilms tumors and genetic alterations in *WT1* and *CTNNB1* analyzed. Chemotherapy induced *MYF6* 165‐fold and several *MYL* and *MYH* genes more than 20‐fold and repressed many genes from cell cycle process networks. Viable tumor cells could be cultivated when patients received less than 8 weeks of chemotherapy but not in two cases with longer treatments. In one case, viable cells could be extracted from a lung metastasis occurring after 6 months of intensive chemotherapy and radiation. Comparison of primary tumor and metastasis cells from the same patient revealed up‐regulation of *RELN* and *TBX2*,*TBX4* and *TBX5* genes and down‐regulation of several *HOXD* genes. Our analyses demonstrate that >8 weeks of chemotherapy can induce terminal myogenic differentiation in *WT1*‐mutant tumors, but this is not associated with volume reduction. The time needed for all tumor cells to achieve the terminal differentiation state needs to be evaluated. In contrast, prolonged treatments can result in genetic alterations leading to resistance.

## Introduction

Wilms tumor (WT), a malignant childhood neoplasm of the kidney, is thought to arise from intermediate mesodermal precursor cells with impaired differentiation potential. Most Wilms tumors have a mixed histology containing blastema, epithelia, and stroma. The stromal‐type variant of Wilms tumor may contain heterotypic cells such as rhabdomyoblasts, fat, cartilage, and bone. These elements are not normally present in the kidney and are likely derived from abnormal mesenchymal differentiation. Constitutional or somatic mutations in the *WT1* gene are found in most stromal‐type tumors, often associated with mutations in the *CTNNB1* gene 1, 2, 3.

Wilms tumors are very efficiently treated by surgery and chemotherapy. In Europe and many other countries, most Wilms tumor patients receive preoperative chemotherapy followed by nephrectomy and risk‐adapted postoperative chemotherapy taking into consideration the remaining histologic features (the Society of Pediatric Oncology (SIOP) protocol); in the United States and a few other countries, patients are treated by upfront surgery and risk‐adapted chemotherapy according to the Children′s Oncology group (COG) (before 2002, the National Wilms Tumor Study group (NWTS)) protocols. Backbone chemotherapy in both protocols consists of vincristine and actinomycin‐D. In high‐risk cases, doxorubicin and tumor bed radiotherapy are often considered. An overall survival rate greater than 90% is reported in cooperative group studies 4, 5, 6.

In the SIOP‐9/GPOH (Gesellschaft für Pädiatrische Onkologie und Hämatologie) trial, it was noticed that stromal and epithelial Wilms tumors showed a poor response to preoperative chemotherapy as measured by tumor volume before and after chemotherapy 7. We have previously reported no volume reduction or even enlargement during preoperative chemotherapy in 24 *WT1*‐mutant Wilms tumors 3. In the NWTS‐4 study, 20% of patients with bilateral Wilms tumor had progressive or nonresponsive disease and these patients were treated for a median of 7 months (2–29 months) with chemotherapy before surgery 8. After the initial chemotherapy regimen, most children received a second regimen and some a third regimen. Histology was rhabdomyomatous or differentiated stromal elements in 14 tumors (36%), but anaplastic tumors were found in four (10.5%) cases 8. Response without shrinkage associated with rhabdomyomatous differentiation in postchemotherapy tumors was also observed by Anderson et al. 9. In addition, a poor radiologic response after preoperative chemotherapy suggestive of resistance was reported for fetal rhabdomyomatous nephroblastoma (FRN) cases 10, 11, 12. The presence of more differentiated tissues in pretreated Wilms tumors was thought to be chemotherapy‐induced 13, 14. We and others have reported that a high percentage of differentiated muscle cells are found in chemotherapy‐treated *WT1*‐mutant tumors, suggesting that cells with these mutations have an intrinsic ability to differentiate in vivo 3, 15, 16. Furthermore, four patients with identical germ line R390X *WT1* mutations developed bilateral Wilms tumors and biopsies before chemotherapy revealed fetal rhabdomyomatous or nephroblastic‐type striated muscle histology 17. The tumors increased during chemotherapy, and additional aggressive therapy was given in all cases. After chemotherapy, myogenin was down‐regulated with concomitant up‐regulation of S‐100 protein, expressed in mature muscle cells 17. Conventional chemotherapy agents target proliferating cells, but differentiated nonproliferating cells are much less affected. It is currently unresolved whether differentiation induced by chemotherapy is reversible.

We show in this report that a significant number of genes associated with terminal myogenic differentiation are induced by chemotherapy and concomitantly a large fraction of cell cycle genes are down‐regulated in *WT1*‐mutant Wilms tumors.

## Patients and Methods

In our international collaboration, we collected Wilms tumor samples from patients with preoperative chemotherapy or untreated tumors. All patients had either tumor‐specific or germ line mutations in *WT1* in different exons. These were used to set up cell cultures and perform gene expression profiles from tumor samples. Treatment regimens of all patients and genetic characteristics of Wilms tumor samples included in this study are listed in Table S1. In this work, we used the nomenclature for the SIOP9/GPOH patients as reported 3.

From patient Wilms1, tumor and relapse samples were available, and the genetics and treatments of this patient have been reported 18. Patient Wilms10 developed a lung metastasis during this study. Clinical details of the patient and the primary tumor were described before 19. The patient had a stage III tumor and was treated according to the NWTSG‐5 protocol with vincristine, actinomycin‐D, and doxorubicin for a total of 6 months including tumor‐ bed radiotherapy. A single lung nodule was detected by routine follow‐up after the end of treatment. This nodule showed a monomorphous rhabdomyomatous histology without anaplasia. After lung surgery, the patient received monthly vincristine and actinomycin‐D for a total of 12 months. Currently, the patient remains in continued complete remission and off chemotherapy 4.5 years after initial diagnosis.

The molecular investigations for the GPOH tumors were approved by the local ethics committees of Heidelberg (31 August 1989), the Saarland (Dr. N. Graf) study nr. 248/13, 104/10, 136/01, and Düsseldorf study nr. 2617. Work with tumors from the Hospital Sant Joan de Déu in Barcelona was approved by the local Institutional Review Board and is in accordance with the principles expressed in the Declaration of Helsinki. All parents of Wilms tumor patients gave a written consent for our work.

### Cell culture

Tumor‐derived cell lines using stem cell MSCG medium and normal cells using WT medium were established by our published protocol 20. Tumor‐derived cell lines were regularly authenticated by their respective genetic alterations in *WT1* and *CTNNB1*.

### Protein analysis

For the SIOP9/GPOH Wilms tumor samples, a method for simultaneous extraction of RNA, DNA, and protein was used as reported 21.

### Gene expression analysis

RNA was isolated from the SIOP tumor samples as described in Ref. 21 and from the other tumor samples with the RNeasy Mini Kit (Qiagen). The Wilms tumor samples used for RNA isolation are summarized in Table S1. Labeling of the RNA, microarray‐hybridization, and processing of the gene expression data was performed essentially as published previously 20. Heat maps were created with “pheatmap” 22 available from CRAN (cran.r‐project.org). Gene expression data can be found at GEO: GSE63616 and GSE102723. To identify biological processes associated with gene expression profiles, the MetaCore software suite (http://thomsonreuters.com/metacore/) was used. The normalized microarray data from up‐ and down‐regulated data sets were uploaded into MetaCore. We selected genes with a minimum fluorescence intensity of 200 in at least one sample. The fold change (fc) and *P*‐values used for data analysis are shown in the individual sections. To study the difference between treated and untreated tumors, we first calculated the mean gene expression levels from the 11 chemotherapy‐treated and the seven untreated Wilms tumor samples. These gene sets were uploaded in MetaCore and analyzed using enrichment analysis of “process networks,” reflecting the content of a MetaCore database that was manually created based on GO processes, pathway maps, and network models of main cellular processes.

## Results

### Induction of a myogenic transcriptional network by preoperative chemotherapy in *WT1*‐mutant Wilms tumors

Here, we analyzed whether induction of muscle cell differentiation is a common feature of chemotherapy‐treated Wilms tumors with different *WT1* mutations. To obtain a more comprehensive insight, we compared the gene expression profiles from 11 preoperatively treated and seven untreated tumor samples.

**Figure 1:**
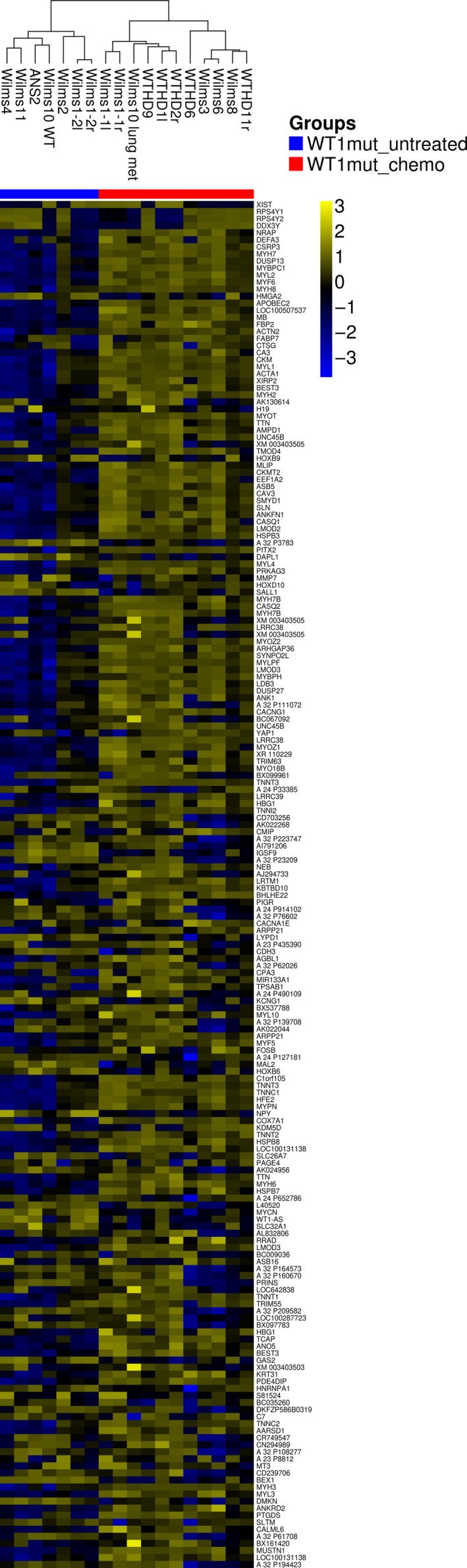
Top 500 genes with the highest coefficient of variation were clustered. The tumor samples with preoperative chemotherapy are indicated in pink color and untreated samples with yellow color. The intensity of expression is shown, and among the genes with the highest coefficient of variation, more genes are up‐regulated than down‐regulated.

**Figure 2:**
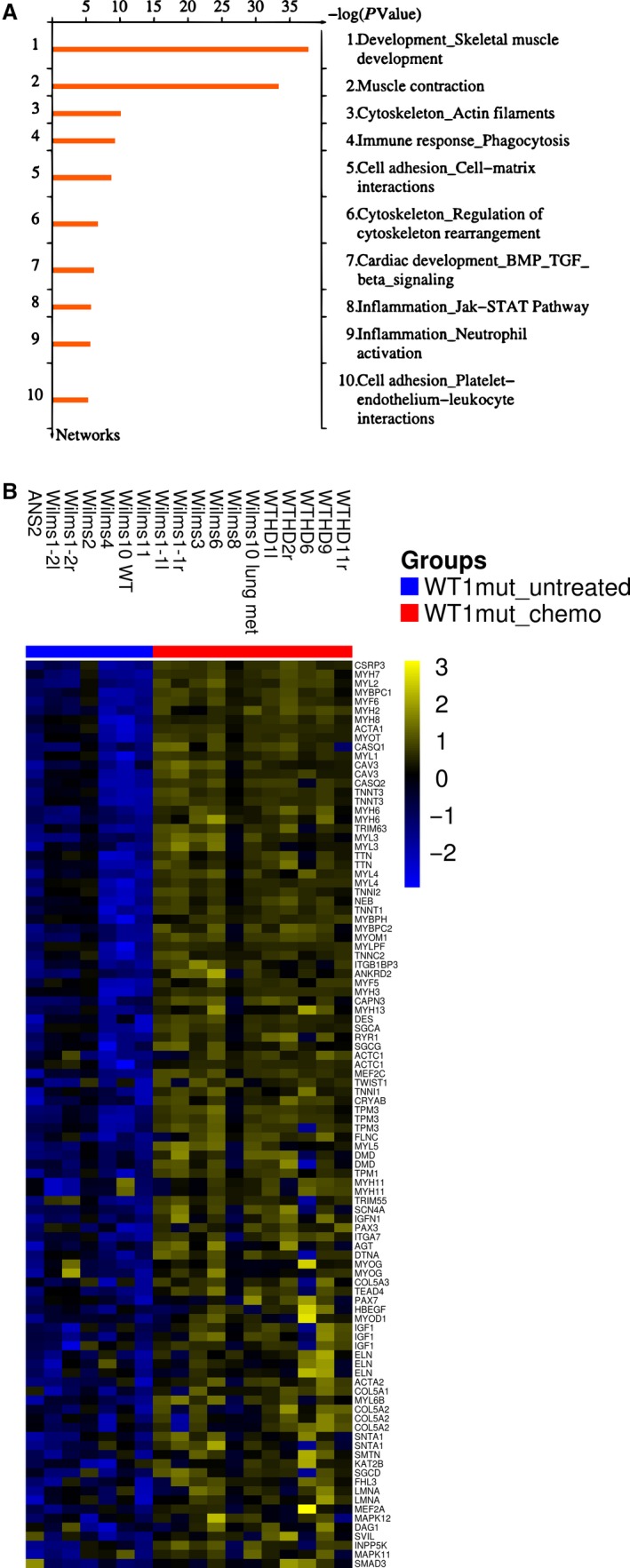
Chemotherapy‐induced genes in the *WT1*‐mutant tumor samples. (A) Ten top process networks. (B) Up‐regulated genes from the top enriched network “skeletal muscle development” are shown. For the heat maps, clustering was performed using Ward's method and Euclidean distance. Colors are yellow for high and blue for low expression.

Hierarchical clustering of 500 genes with the highest coefficient of variation revealed specific clusters of chemotherapy‐treated and untreated Wilms tumors (Fig. 1). We identified 1452 up‐ and 1613 down‐regulated genes (fc >1.5, *P*‐value 0.05) between the cohorts of untreated and treated tumor samples. The 10 top ranking networks for chemotherapy‐induced genes are shown in Figure 2A. Enrichment of the process network “Development_Skeletal muscle development” was most significant (mean FDR (false discovery rate) 5.5e‐36), with 80 of 144 genes showing increased expression, followed by process network “Muscle contraction” (mean FDR 7.8e‐32). The mean fold increase in two genes associated with terminal muscle differentiation was 4.3 for *MYOG* and 165 for *MYF6*. Various myosin heavy‐ and light‐chain genes were up‐regulated more than 20‐fold (Table S2). A heat map of genes from the process network “Development_Skeletal muscle development” shows some variation of gene expression levels between individual tumors (Fig. 2B). However, most genes of this network are up‐regulated in all 11 chemotherapy‐treated Wilms tumors, regardless of the therapeutic regimen (Table S1). The up‐regulation of *MYOG* and *ACTC1* in the relapsed but untreated Wilms1‐2r could be related to the chemotherapy that this patient received 1 year before surgery of the first set of tumors. In addition, a significant enrichment of the process network, “Cardiac development_BMP_TGFbeta signaling” (mean FDR 6.4e‐05), was identified with increased expression of genes expressed in immature and mature cardiomyocytes including *TNNT2, TNNC1*,* TNNC1*,* MYL2*,* MYH6*,* MYH7*,* MEF2C*,* IRX4,* and *ACTC1*. These results show that chemotherapy caused an induction of genes from multiple myocyte differentiation programs in *WT1*‐mutant Wilms tumor cells in vivo.

**Figure 3:**
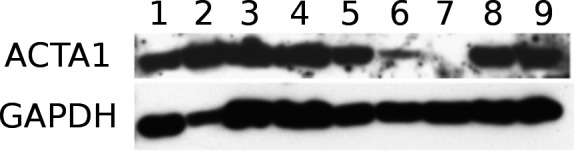
Expression of ACTA1 in tumor samples. Lanes 2–6, 8, and 9 contain tumor protein extracts from samples obtained after chemotherapy. 2: WTHD11r; 3: WTHD6; 4: WTHD2r; 5: WTHD1 l; 6: WTHD9; 8: WTHD10; 9:WTHD14. Lane 1 is an extract from fetal muscle as control. Sample 7 is from the untreated ANS tumor. GAPDH was used a loading control.

We selected the *ACTA1* gene which is 60‐fold up‐regulated in chemotherapy‐treated Wilms tumors to check whether ACTA1 protein expression levels correlate with gene expression levels. ACTA1 protein was analyzed in seven chemotherapy‐treated tumor samples and all are positive (Fig. 3, lanes 2–5, lane 8 + 9); one tumor shows a low level (Fig. 3, lane 6), confirming the gene expression data. A protein preparation of an untreated Wilms tumor (ANS2) was analyzed as control and ACTA1 was undetectable (Fig. 3, lane 7).

**Figure 4:**
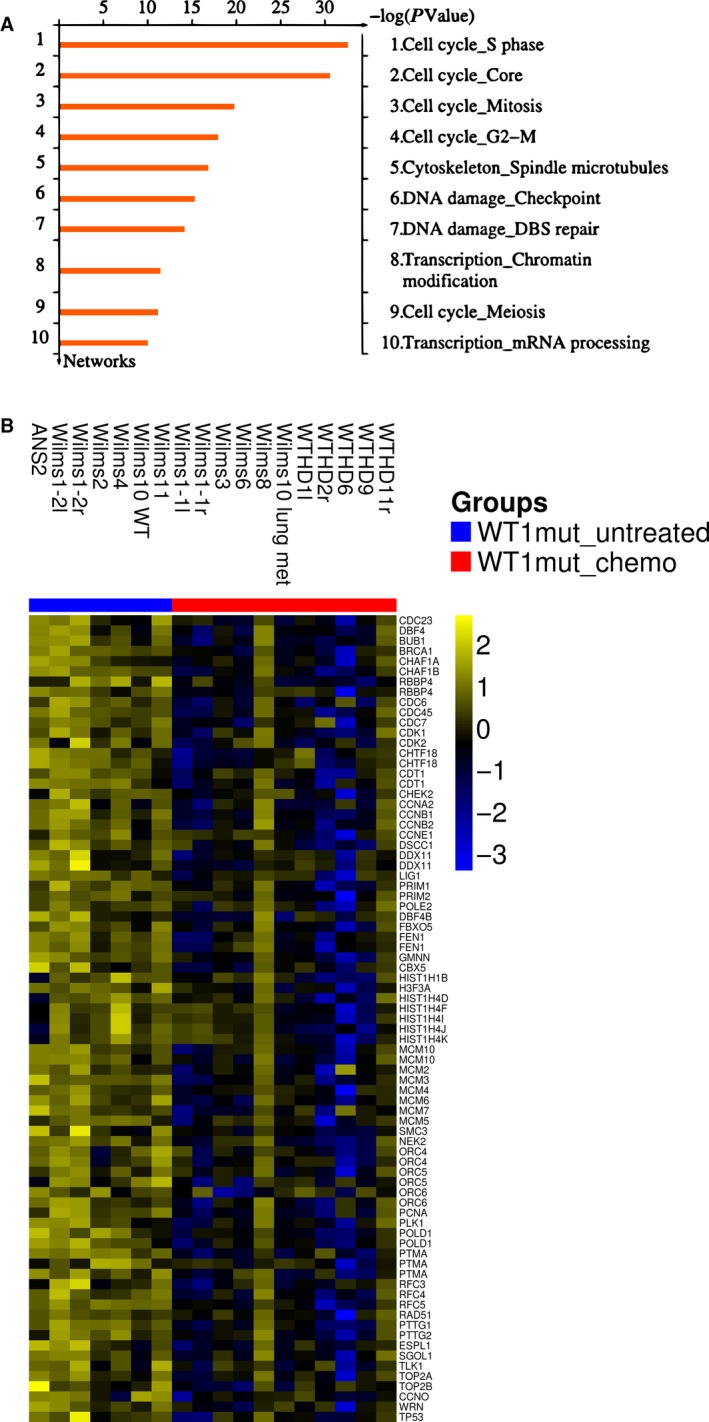
Chemotherapy‐repressed genes in *WT1*‐mutant tumor samples. (A) Ten top process networks. (B) Down‐regulated genes from the network “cell cycle S‐phase” are shown. For the heat maps, clustering was performed using Ward's method and Euclidean distance. Colors are yellow for high and blue for low expression.

The analysis of down‐regulated genes identified many process networks related to the cell cycle. The 10 top ranking networks are shown in Figure 4A. Here, the network “Cell cycle_S phase” is most significant (mean FDR 6.6 e‐31) and the individual expression‐level differences between tumor samples are visualized in a heat map (Fig. 4B). The list of genes of this network with the mean fc is shown in Table S3. It is important to note that besides “Cell cycle_S phase” and “Cell cycle_Core, ”“Cell cycle_Mitosis” and “Cell cycle_G2‐M” were identified as significantly enriched. Several genes regulating the S and G2M phases of the cell cycle were among the down‐regulated genes, for example, *CDK1*,* CDK2,* and cyclins. In addition, many genes involved in DNA replication and repair and in chromatin modification were down‐regulated as well.

### Wilms tumor‐derived cell lines as model system to study the effects of chemotherapy in Wilms tumor patients with mutant *WT1* genes

We have successfully established cell lines from 11 *WT1‐*mutant Wilms tumors from patients having received 4‐ to 8‐week preoperative chemotherapy or were untreated before surgery (Table S1). To study whether chemotherapy can induce terminal/irreversible muscle cell differentiation, we tried to set up cell cultures from two tumors treated for more than 8 weeks of preoperative chemotherapy. In both cases, Wilms12 and Wilms13, we were unable to establish tumor‐derived cell lines, and Wilms12 cell cultures showed individual cells with skeletal muscle morphology that did not adhere to the dish and did not proliferate (Fig. S1A). Patient Wilms12 had a germ line heterozygous *WT1* mutation, and the primary tumor sample was homozygous for this mutation (Fig. S2B). Cell cultures with epithelioid morphology could be cultivated using WT medium (Fig. S1B), and the isolated DNA showed a heterozygous *WT1* mutation (Fig. S2C). Furthermore, although the primary tumor carried a *CTNNB1* mutation (Fig. S3B), the cells cultivated in WT medium were wild type (Fig. S3C). These results demonstrate that the epithelioid cells were normal somatic cells. Finally, no viable cells from the tumor of patient Wilms13 could be propagated. These two cases suggest that prolonged chemotherapy had induced terminal muscle differentiation with loss of in vitro growth potential.

Patient Wilms10 with a tumor‐specific homozygous *WT1* deletion within a heterozygous 11p13 deletion and a tumor‐derived cell line (Wilms10T) have been reported. A lung nodule was detected at a routine follow‐up after 6 months of treatment and removed by surgery. The stromal predominant Wilms tumor had no anaplasia, and immunohistochemistry revealed the presence of Ki67‐positive cells, that is, proliferating cells in the metastasis (Fig. S4). Importantly, we could establish a metastasis‐derived cell line from this patient (Wilms10M).

**Figure 5:**
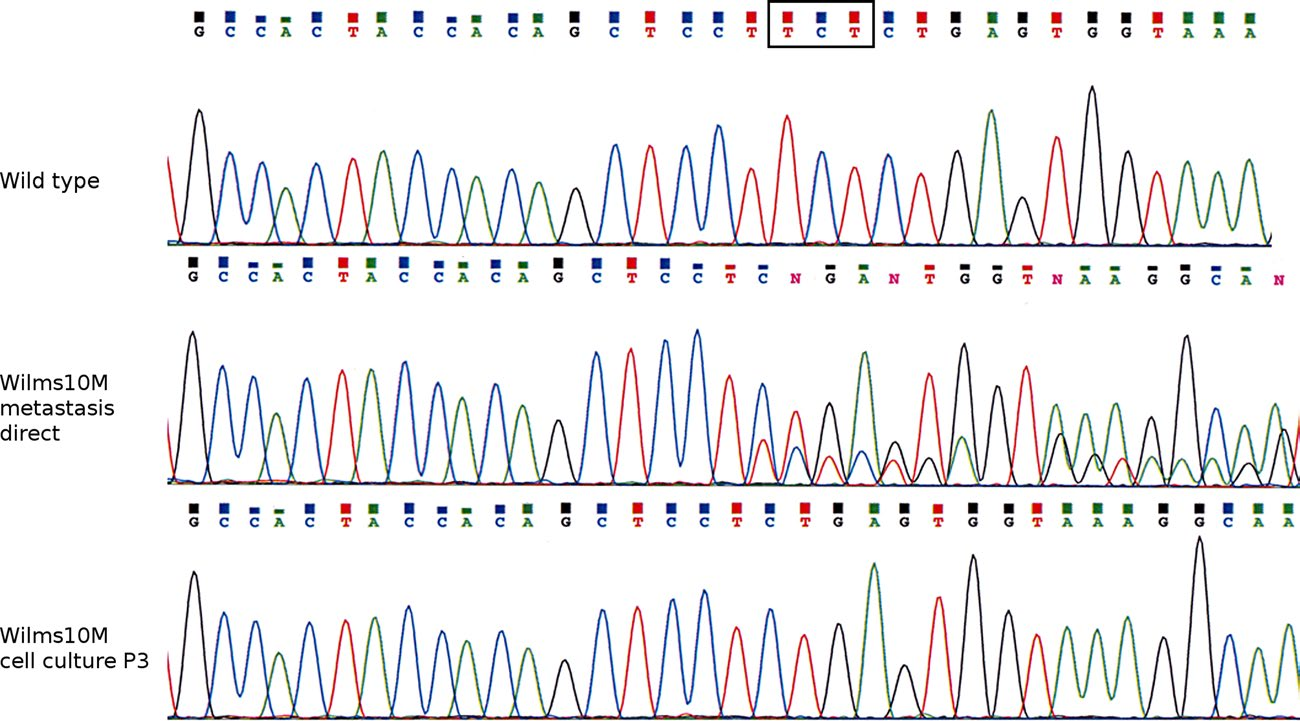
Genetic analysis of the *CTNNB1* gene in Wilms10 metastasis. The top panel shows the wild‐type *CTNNB1* sequence. The sequence of the three nucleotide deletion is boxed; the middle panel shows the sequence of DNA isolated directly from the lung metastasis, and the mixed pattern starting at the position of the deletion is seen with more mutant sequence; the bottom panel shows the sequence of the metastasis cell culture DNA, only the homozygous deletion of three nucleotides is seen.

DNA isolated from the lung metastasis had the same somatic homozygous *WT1* deletion within the heterozygous 11p13 deletion and the uniparental disomies (UPD) in 1p, 3p, and 11p15 as the primary kidney tumor. In addition, the analysis of the lung metastasis revealed the same homozygous p.S45Δ *CTNNB1* mutation as the primary Wilms10T cell line (Fig. 5). Wilms10M cells carried a homozygous p.S45Δ *CTNNB1* mutation (Fig. 5). Therefore, the lung metastasis and its derived cell culture were originated from a clone of cells within the primary tumor that carried the homozygous p.S45Δ mutation, whereas the bulk primary tumor DNA was heterozygous for another *CTNNB1* mutation. The gene expression analysis of the lung metastasis in vivo indicated that most cells had acquired a terminal skeletal muscle differentiation state (see Figs 2 and 4), but the cell culture data indicate that a subpopulation of Wilms10 tumor cells was still actively proliferating and had developed a drug‐resistant phenotype during treatment.

### Genetic properties of lung metastasis cells from patient Wilms10

To gain insight into the mechanisms of acquired drug resistance, we investigated the genetic properties of metastatic Wilms10M cells. The cell culture from the primary tumor had a normal karyotype, whereas the Wilms10M cells showed an aberrant karyotype: 47,XX,+8[22]/47,idem,add(1)(p36)[4]. ArrayCGH analysis identified two identical deletions on chromosome 1p in Wilms10T and Wilms10M cell lines (Figs S5 and S6), the identical chromosome 11p13 alteration (Fig. S7) and two germ line CNVs on chromosomes 14 and 15, confirming their authenticity (Figs S8 and S9). Furthermore, the metastasis‐specific trisomy 8 was confirmed (Fig. S10). These data show that the lung metastasis cells acquired a gain of chromosome 8 not present in the primary tumor.

Next, we compared the gene expression profile of cultured Wilms10M and Wilms10T cells. Using stringent analysis parameters (FDR of 0.1 and fc of 1.5), we identified 80 up‐ and 29 down‐regulated genes in Wilms10M cells from the lung nodule. The highest up‐regulated gene was *RELN* (397‐fold), which encodes a secreted extracellular matrix protein thought to be involved in cell–cell interactions, cell positioning, and migration. Several members of the *TBX* gene family were also expressed at much higher levels in Wilms10M cells, for example, *TBX5* (177‐fold), *TBX4* (138‐fold), and *TBX2* (14‐fold). The expression of *TBX2* was shown to block muscle cell differentiation and to promote proliferation of rhabdomyosarcoma cells. A table of these significantly up‐regulated genes (FDR of 0.1) is shown as Table S4. Among the down‐regulated genes, we identified several *HOXD* genes, for example, *HOXD1, D3, D4,* and *D8* (Table S5).

For a more comprehensive overview of the gene expression data, the up‐ and down‐regulated genes were analyzed separately with a less stringent cutoff (uncorrected *P*‐value 0.05, fc of 2). The up‐regulated gene set revealed a significant enrichment of the network “Development, negative regulation of STK3/4(Hippo) pathway and positive regulation of YAP/TAZ function” (FDR 7.7 e‐5), where 15 of 62 pathway genes show a higher expression in Wilms10M cells. This is relevant as activation of YAP is elevated in embryonal rhabdomyosarcoma (ERMS) and may be associated with a differentiation block.

## Discussion

A comprehensive approach was used to identify key mechanisms responsible for the chemotherapy response of *WT1‐*mutant Wilms tumors. We established tumor‐derived cell cultures, analyzed evolution of genetic alterations, and compared gene expression profiles of untreated and treated *WT1‐*mutant Wilms tumors. Although previously it was postulated that chemotherapy induces a more differentiated skeletal muscle phenotype in certain Wilms tumors, a direct comparison of *WT1*‐mutant Wilms tumor samples before and after chemotherapy using whole transcriptome analysis has not been reported. Studies using gene expression data can provide an unbiased view of how *WT1*‐mutant Wilms tumors respond to chemotherapy. Here, we show that a more mature skeletal muscle differentiation is induced by chemotherapy in all *WT1*‐mutant Wilms tumors. Skeletal muscle development is regulated by a family of transcription factors termed myogenic regulatory factor family (MRF), with MYOD and MYF5 having important roles in the determination of the myogenic cell lineage, whereas MYOG and MYF6 are associated with terminal skeletal muscle differentiation and homeostasis of myofibers 23, 24.The induction of a myogenic transcriptional network with the up‐regulation of 80 genes of the skeletal muscle pathway including *MRF4* and *MYOG* indicates that the stage of terminal skeletal muscle differentiation was achieved 24. It is known that terminally differentiated muscle cells are postmitotic, and we were unable to establish tumor‐derived cell lines from Wilms12 and Wilms13 tumors that had received more than 8 weeks of preoperative chemotherapy. Furthermore, the induction of skeletal muscle cell differentiation is specific for the tumor cells as normal (*CTNNB1* wild‐type) cells from Wilms12 propagated in tissue culture did not show any evidence of muscle differentiation.

In contrast to Wilms12 and 13 cases, the patient from whom Wilms10T cells were established developed a lung metastasis despite extended postoperative chemotherapy. It was unexpected that viable cells from the lung metastasis could be cultivated in vitro as the gene expression profile of the metastasis clustered with the chemotherapy‐treated Wilms tumor samples, indicating that most cells had differentiated. The primary untreated tumor and the metastasis had three identical genetic aberrations in common, two tumor‐specific deletions on chromosome 1p and the 11p13 alteration, suggesting that these cells in the primary tumor contributed to the development of the lung metastasis. The trisomy 8 in the metastasis was not present in the primary Wilms10 and in cultured Wilms10T cells, and this was likely induced by chemotherapy. The Wilms10M cells showed increased expression of genes that can block muscle differentiation, such as *TBX2* 25 and up‐regulation of the YAP/TAZ pathway. This is a significant observation as it was shown that normalization of YAP activity in *YAP1* mutation‐driven ERMS‐like tumors in mice resulted in regression of tumors 26. This genetic evolution and up‐regulation of a set of genes could have caused the failure of chemotherapy‐induced terminal skeletal muscle differentiation in these cells. Chemotherapy‐treated *WT1*‐mutant Wilms tumors show a strong down‐regulation of many cell cycle genes. This is a rather common response to chemotherapy. However, in the context of *WT1*‐mutant Wilms tumors, this is of special importance. Several cyclins that are regulatory subunits of cell cycle kinases (*CCNA*,* CCNA2*,* CCNB1*,* CCNB2, CCNE1*) and several catalytic subunits of *CDK1* and *CDK2* were down‐regulated. The *MYOD* gene is inactivated through serine‐200 phosphorylation by active CDK2‐cyclinE kinases 27. Therefore, down‐regulation of cell cycle kinases may result in activation of MYOD1 promoting cell cycle exit and muscle cell differentiation. This potential novel molecular mechanism for the induction of terminal muscle cell differentiation with concomitant loss of malignant growth potential of *WT1*‐mutant Wilms tumor cells will require final functional confirmation. Here, we show that a longer preoperative chemotherapy of *WT1*‐mutant Wilms tumor patients can be associated with the development of terminally differentiated skeletal muscle cells, which are unable to proliferate in vitro in two patients. However, our data with metastatic Wilms10M cells show that this response is more complex as preexisting or acquired genetic alterations can affect tumor cell behavior. The up‐regulation of genes that can block myogenesis and/or genes that can activate YAP/TAZ signaling in Wilms10M cells suggests a potential mechanism of resistance to chemotherapy‐induced differentiation.

It has been established that *WT1‐*mutant Wilms tumors do not show volume reduction after chemotherapy 3. This is explained by the fact that skeletal muscle cell differentiation is induced rather than apoptotic cell death. Thus, a lack of volume reduction in *WT1‐*mutant tumors does not indicate chemotherapy resistance. It was reported that patients with bilateral Wilms tumors, who did not respond to chemotherapy by volume reduction during preoperative chemotherapy, were treated for extended periods of time and often with additional anticancer drugs 8. Some of these tumors showed skeletal muscle differentiation after prolonged therapy, but also several of the tumors had anaplastic histology, possibly induced by the treatment regimens. It is a clinical goal to remove the tumor with nephron‐sparing surgery to conserve functional kidney tissue. These observations lead to a prospective multicentric treatment study where patients were treated with three drug induction chemotherapy for 6 or 12 weeks based on radiographic response before surgery 28. The trial (AREN0534) showed that a three‐drug preoperative chemotherapy and surgical resection within 12 weeks and histology adapted postoperative therapy resulted in improved event‐free and overall survival and preservation of renal parenchyma. This is important in the context of our previous analysis of children with germ line truncation *WT1* mutations demonstrating that 52% of them develop bilateral Wilms tumors 29. It would be interesting to establish imaging methods that can determine the degree of muscle differentiation in tumors from patients with germ line *WT1* mutations rather than measure solely the radiologic response. Ultimately, this could lead to a risk‐adapted therapy protocol sparing the children with *WT1*‐mutant tumors from more intensive chemotherapy and increased chances to develop resistance to skeletal muscle differentiation.

Chemotherapy induces predominantly skeletal muscle cell differentiation in *WT1*‐mutant Wilms tumors, although adipogenic and osteogenic differentiation can also be observed in some tumors and in cell lines 20. Our established *WT1*‐mutant Wilms tumor‐derived cells can be propagated in vitro in an undifferentiated state and can be induced to differentiate in vitro 20. These cell culture model systems will help to analyze whether efficient myogenic differentiation can be induced with individual chemotherapeutic agents or whether specific combinations are required. In addition, these Wilms tumor‐derived cell lines with mutant *WT1* genes are a valuable tool to identify less toxic agents that can induce more efficiently terminal skeletal muscle cell differentiation.

## Conflict of Interest

None declared.

## Supporting information


**Figure S1.** Culturing of cells from Wilms12 tumor.
**Figure S2**. Sequencing of *WT1* exon9 from case Wilms12.
**Figure S3**. Sequencing of *CTNNB1* exon3 from case Wilms12.
**Figure S4**. Wilms10 lung metastasis immunohistochemistry.
**Figure S5**. aCGH analysis of Wilms10T and Wilms10M cells.
**Figure S6**. aCGH analysis of Wilms10T and Wilms10M cells.
**Figure S7**. aCGH analysis of Wilms10T and Wilms10M cells.
**Figure S8**. aCGH analysis of Wilms10T and Wilms10M cells.
**Figure S9**. aCGH analysis of Wilms10T and Wilms10M cells.
**Figure S10**. aCGH analysis of Wilms10T and Wilms10M cells.

**Table S1.** Clinical and genetic information on tumor samples.

**Table S2.** List and mean fc of up‐regulated genes in chemotherapy treated *WT1* mutant tumors.

**Table S3.** List and mean fc of down‐regulated genes in chemotherapy treated *WT1* mutant tumors.

**Table S4.** Genes up‐regulated in Wilms10 metastasis cells (FDR 0.1).

**Table S5.** Genes down‐regulated in Wilms10 metastasis cells (FDR 0.1).
