# Supplementary material for: Chemotherapy and terminal skeletal muscle differentiation in WT1‐mutant Wilms tumors
Source: Cancer Med. 2018 Mar 15;7(4):1359–68. doi: 10.1002/cam4.1379 (PMC5911586; doi:10.1002/cam4.1379)
Supplement: Supplementary file 1 — Figure S1. Culturing of cells from Wilms12 tumor. Figure S2. Sequencing of WT1 exon9 from case Wilms12. Figure S3. Sequencing of CTNNB1 exon3 from case Wilms12. Figure S4. Wilms10 lung metastasis immunohistochemistry. Figure S5. aCGH analysis of Wilms10T and Wilms10M cells. Figure S6. aCGH analysis of Wilms10T and Wilms10M cells. Figure S7. aCGH analysis of Wilms10T and Wilms10M cells. Figure S8. aCGH analysis of Wilms10T and Wilms10M cells. Figure S9. aCGH analysis of Wilms10T and Wilms10M cells. Figure S10. aCGH analysis of Wilms10T and Wilms10M cells. [file CAM4-7-1359-s001.pdf]

Support Figures 1-10

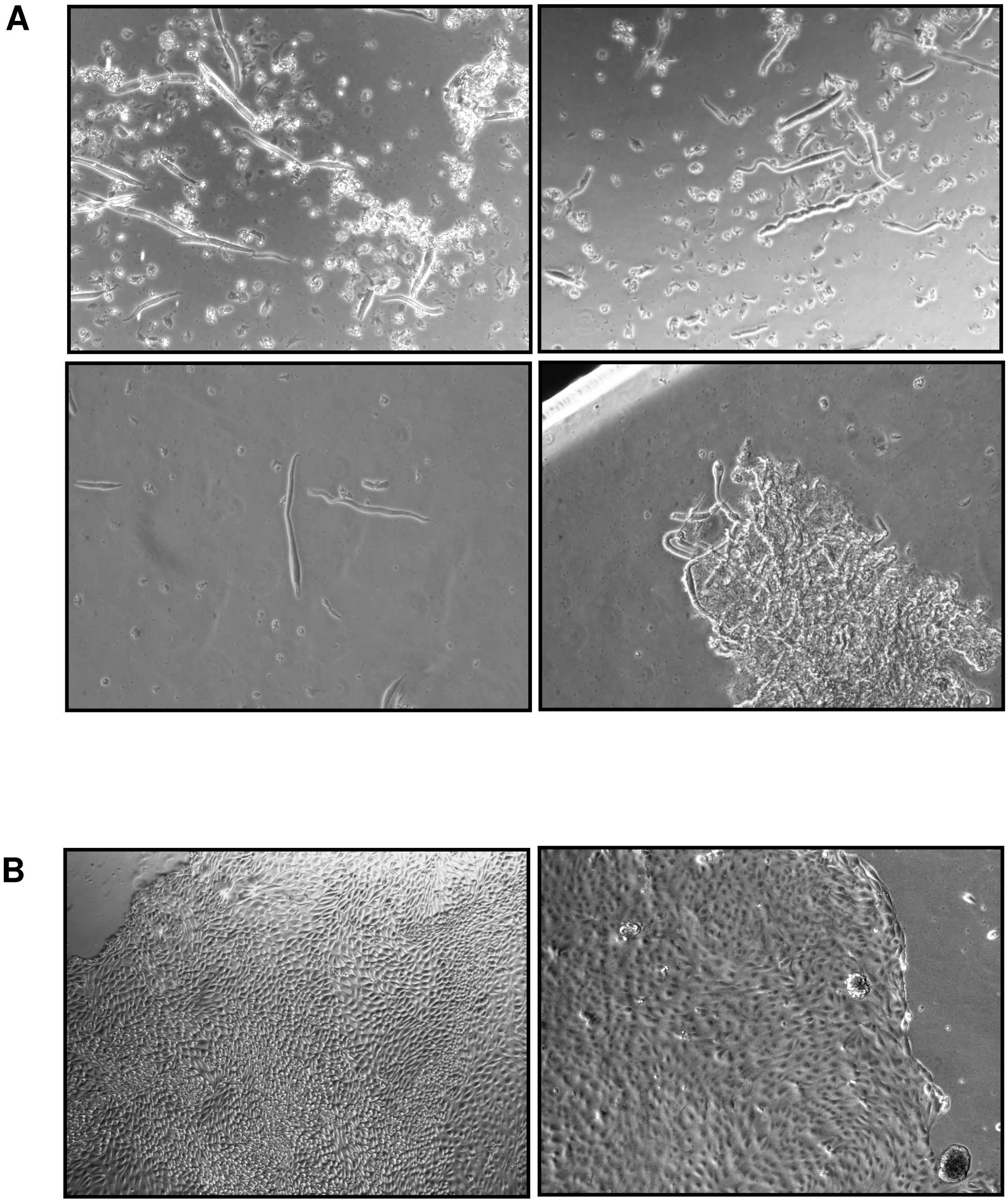

Figure 1. Culturing of cells from Wilms12 tumor
A) Tumor was minced with scalpel and cells were seeded in MSCGM. Individual single long skeletal muscle cells were observed that did not attach. No cells adhered to the flask from the tumor clump. **B)** cells from adjacent normal kidney cultured in WT medium adhered to the dish and formed large colonies with epitheloid morphology and structures resembling S shaped bodies.

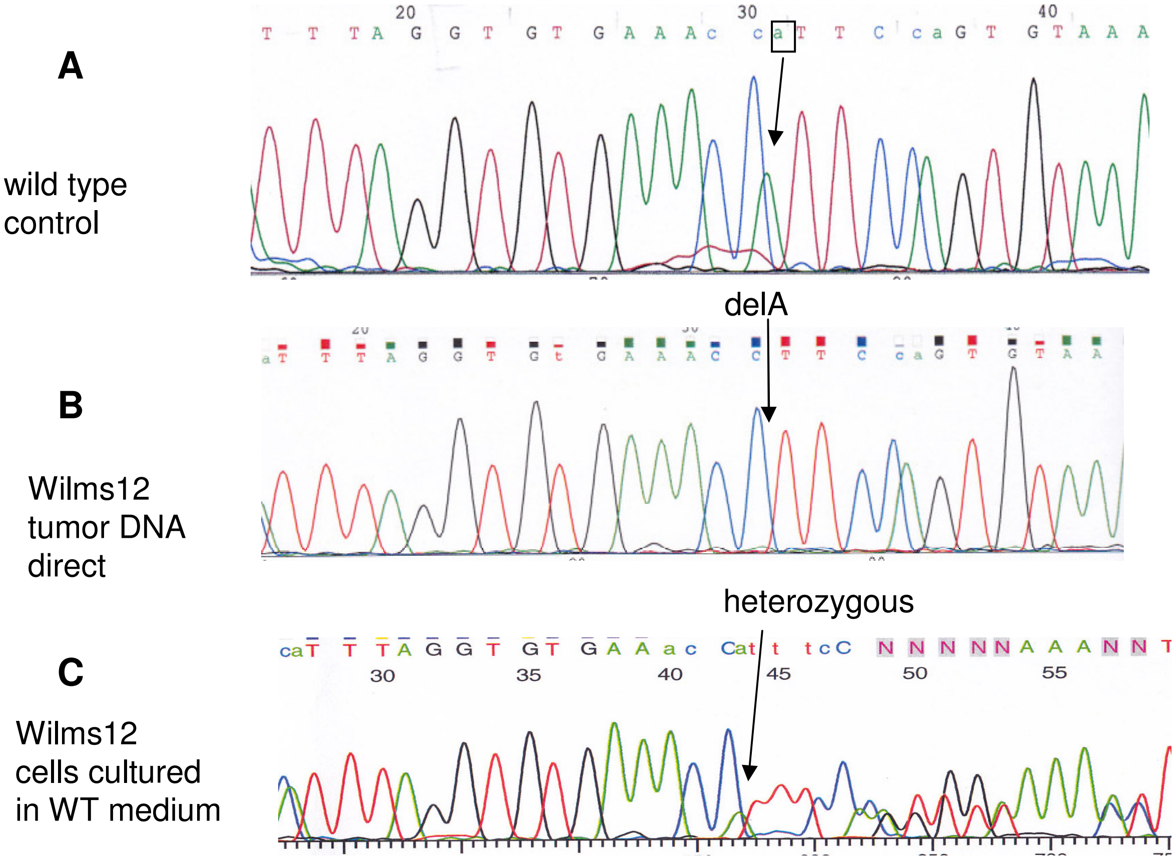

Figure 2. Sequencing of *WT1* exon9 from case Wilms12
A) Normal *WT1* exon9 sequence **B)** DNA isolated directly from the tumor shows a homozygous deletion of 1 nucleotide marked with delA. **C)** DNA isolated from cells cultured in WT medium as shown in supplementary Figure 1B shows a heterozygous deletion of 1 nucleotide, same as in the patients blood DNA.

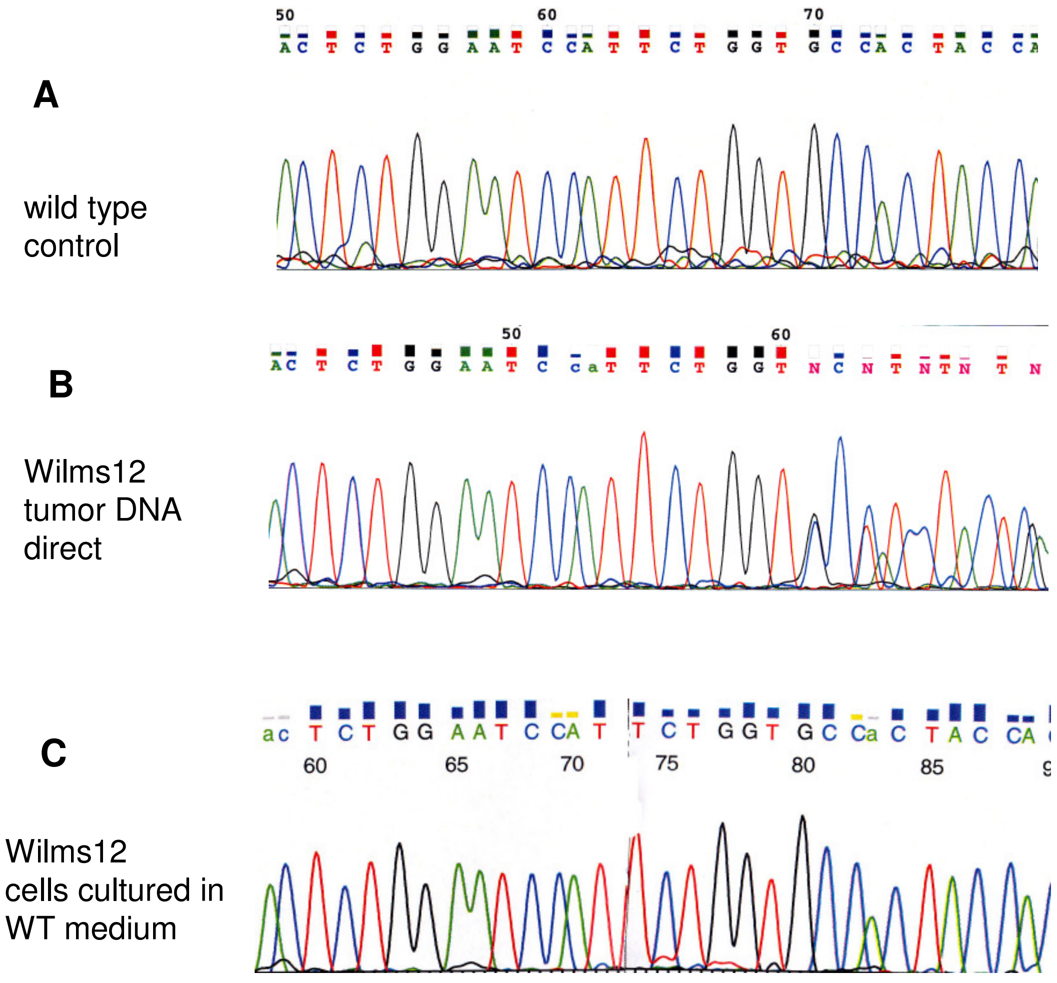

Figure 3. Sequencing of *CTNNB1* exon3 from case Wilms12
A) Normal control, *CTNNB1* exon3 sequence **B)** DNA isolated directly from the tumor shows a heterozygous deletion of 15 nucleotides, resulting in deletion of amino acids Ala39-Ala43. **C)** DNA isolated from cells cultured in WT medium as shown in supplementary Figure 1B shows a wild type sequence.

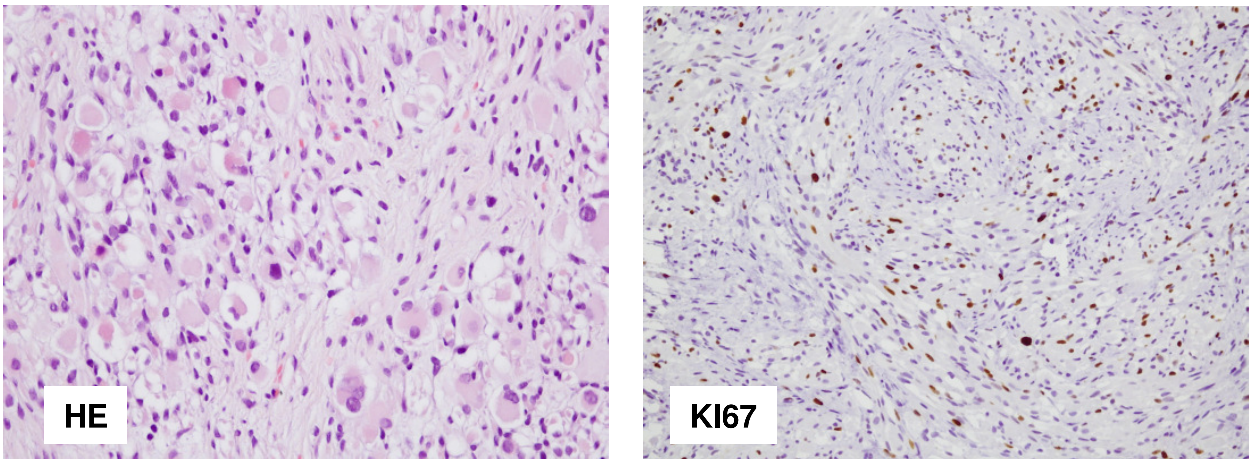

Figure 4. Wilms10 lung metastasis immunohistochemistry
Left: HE stained slide shows the rhabdomyomatous histology. Right: individual Ki67 positive cells are still present.

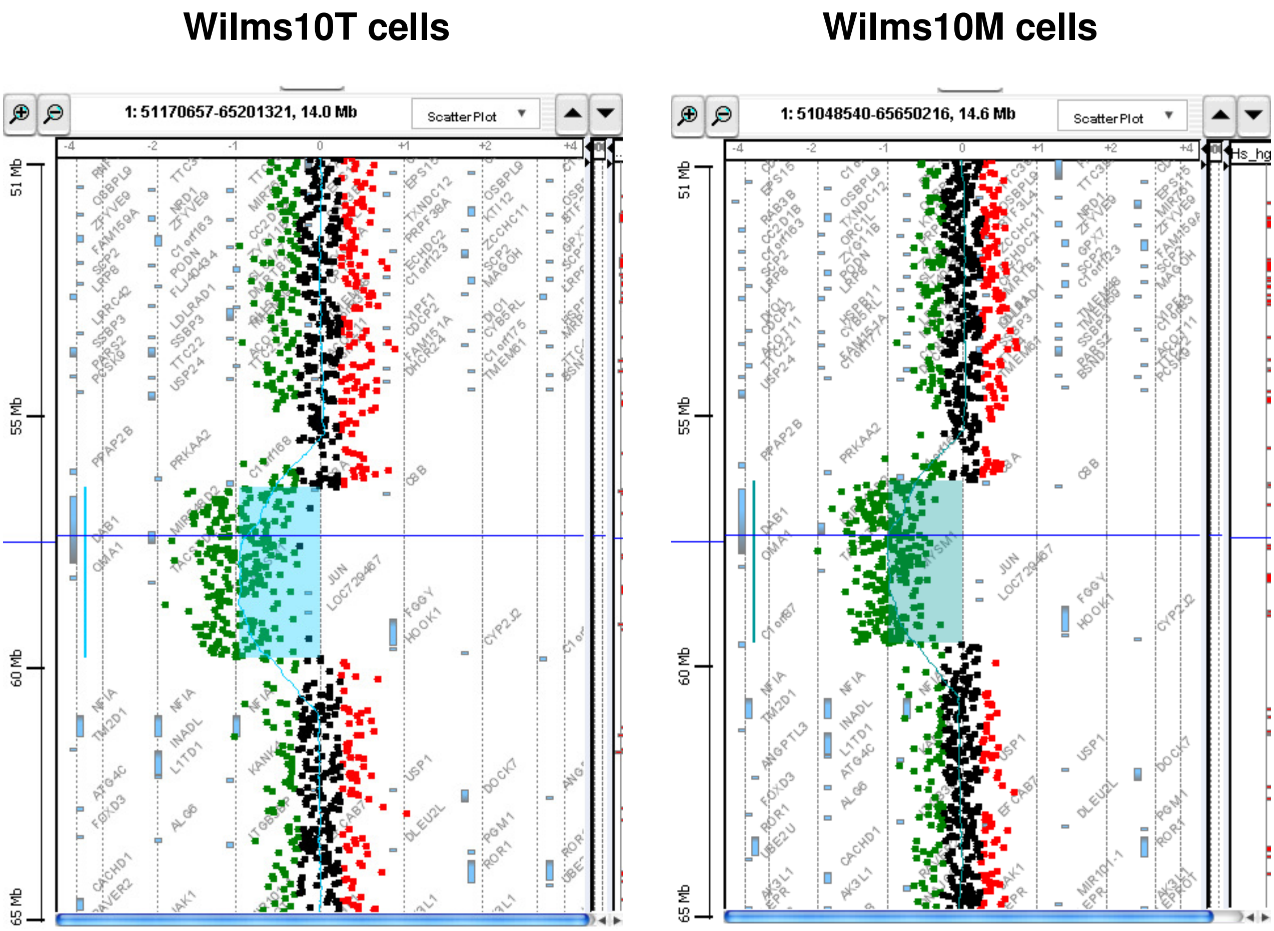

Figure 5. aCGH analysis of Wilms10T and Wilms10 M cells.
Both cell cultures have an Identical deletion in 1p32-p32.1 of a size of 3.2Mb and containg the following genes: *C8B, C8A, DAB1, OMA1, TACSTD2*, *MYSM1, JUN, FGGY, HOOK, CYP2J2* and *C1ORF87*. This deletion is not present in the germline

Wilms10T cells **Wilms10M cells**

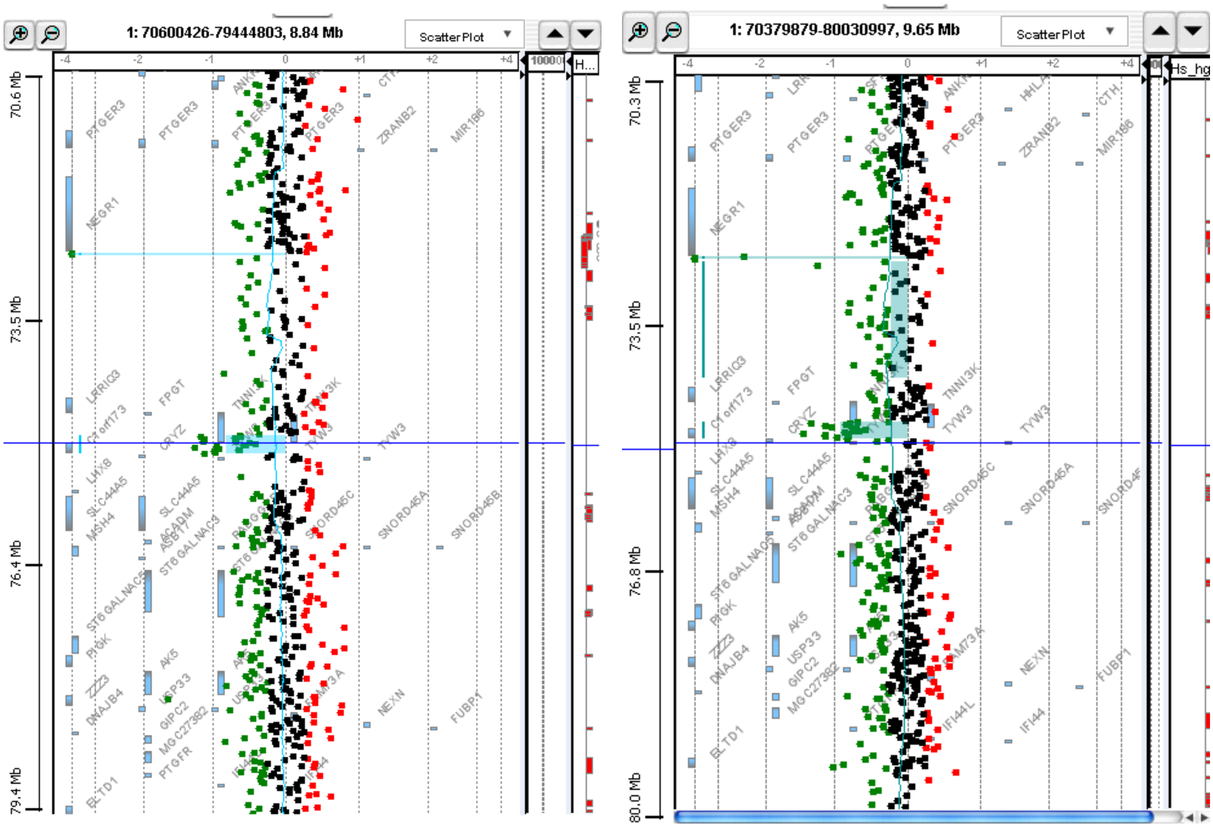

Figure 6. aCGH analysis of Wilms10T and Wilms10 M cells.
Both cell cultures have an Identical deletion in 1p31.1 of a size of 203kb and containg the following genes: *FPGT-TNNI3K* and *ERIC3/C1ORF173*. This deletion is not present in the germline

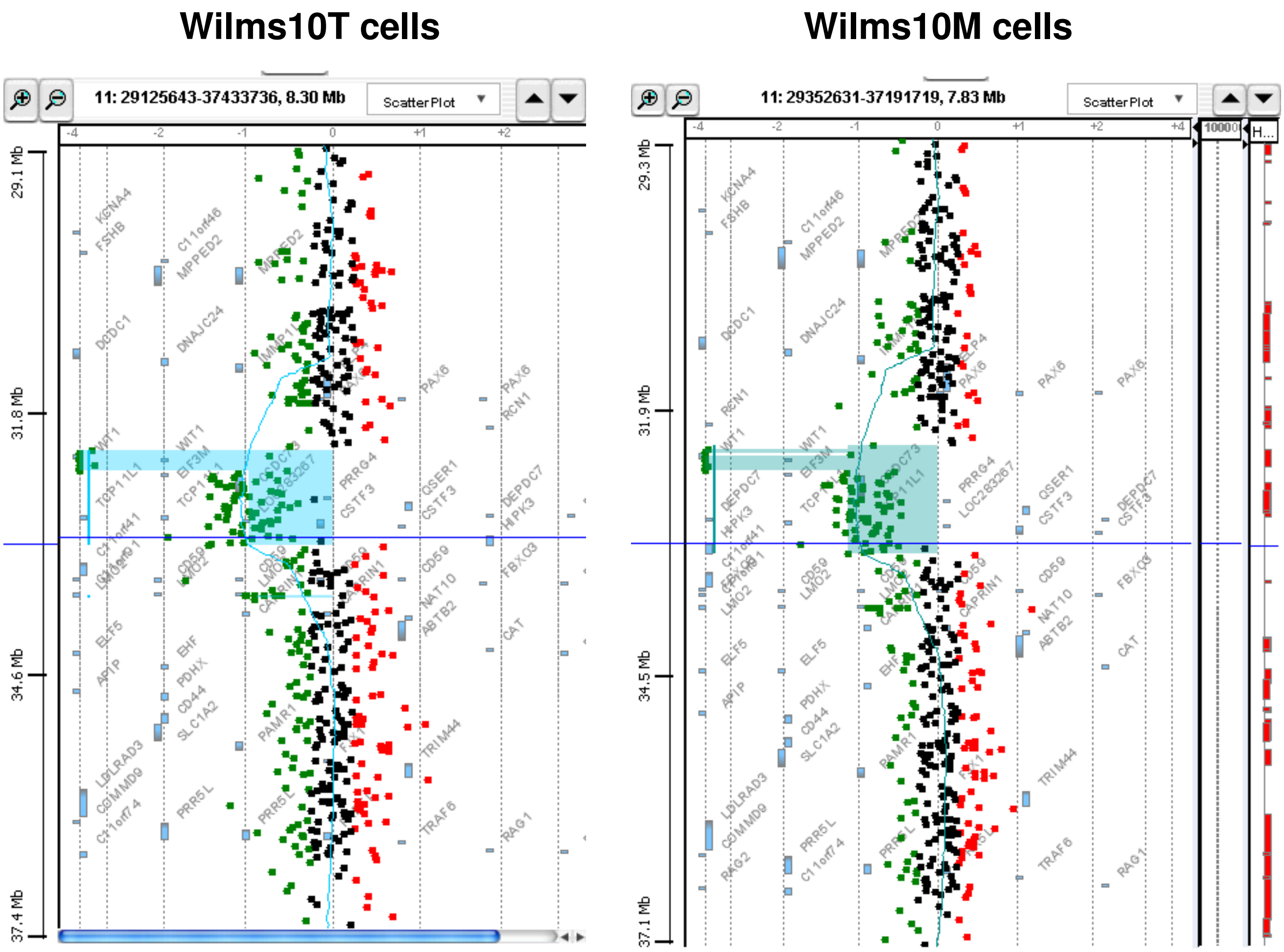

Figure 7. aCGH analysis of Wilms10T and Wilms10 M cells.
Both cell cultures have an identical homozygous deletion of *WT1* within a heterozygous 11p13 deletion, not present in the germline

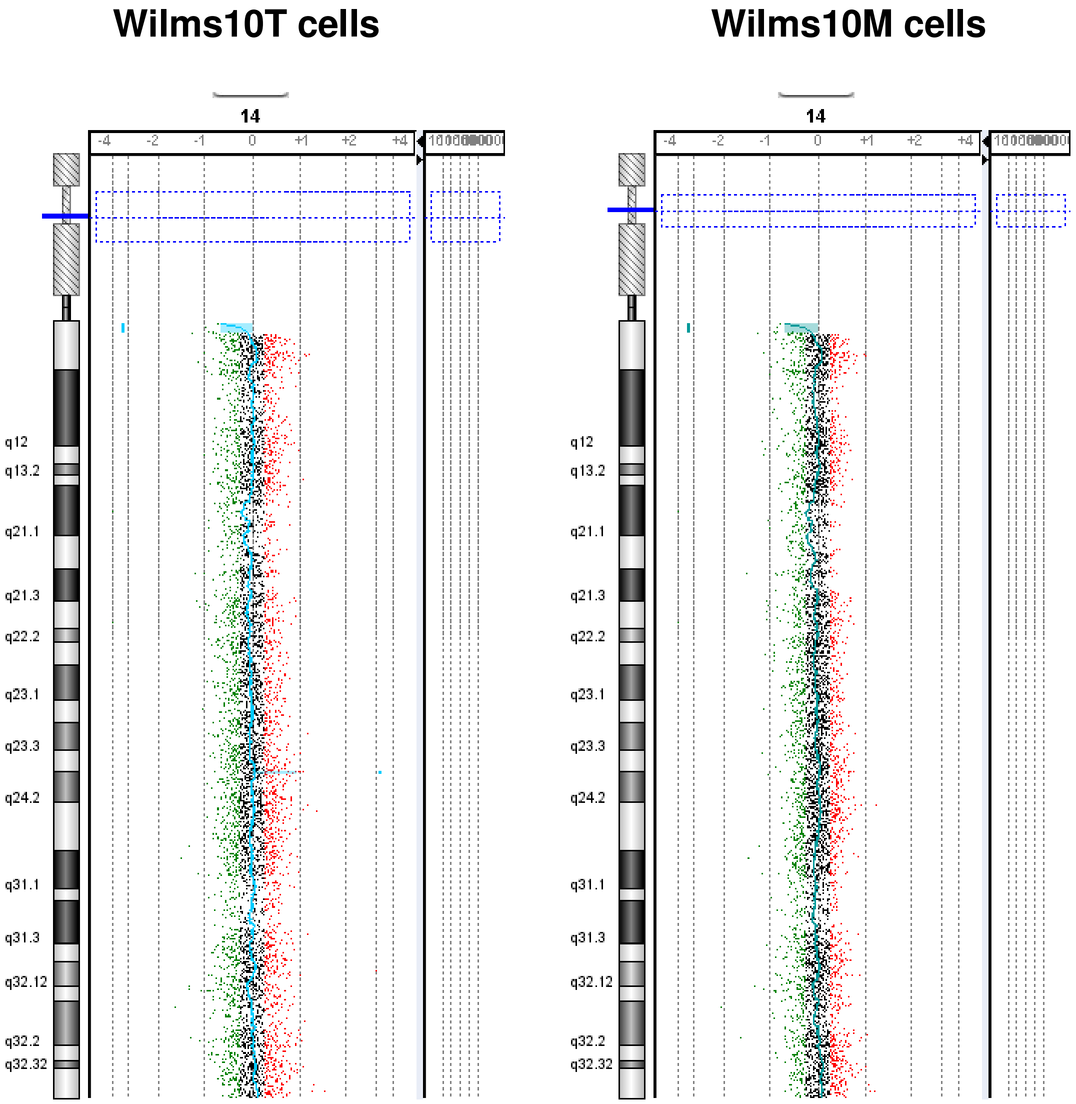

Figure 8. aCGH analysis of Wilms10T and Wilms10 M cells.
Both cell cultures have an identical germ line deletion in 14q, a known CNV

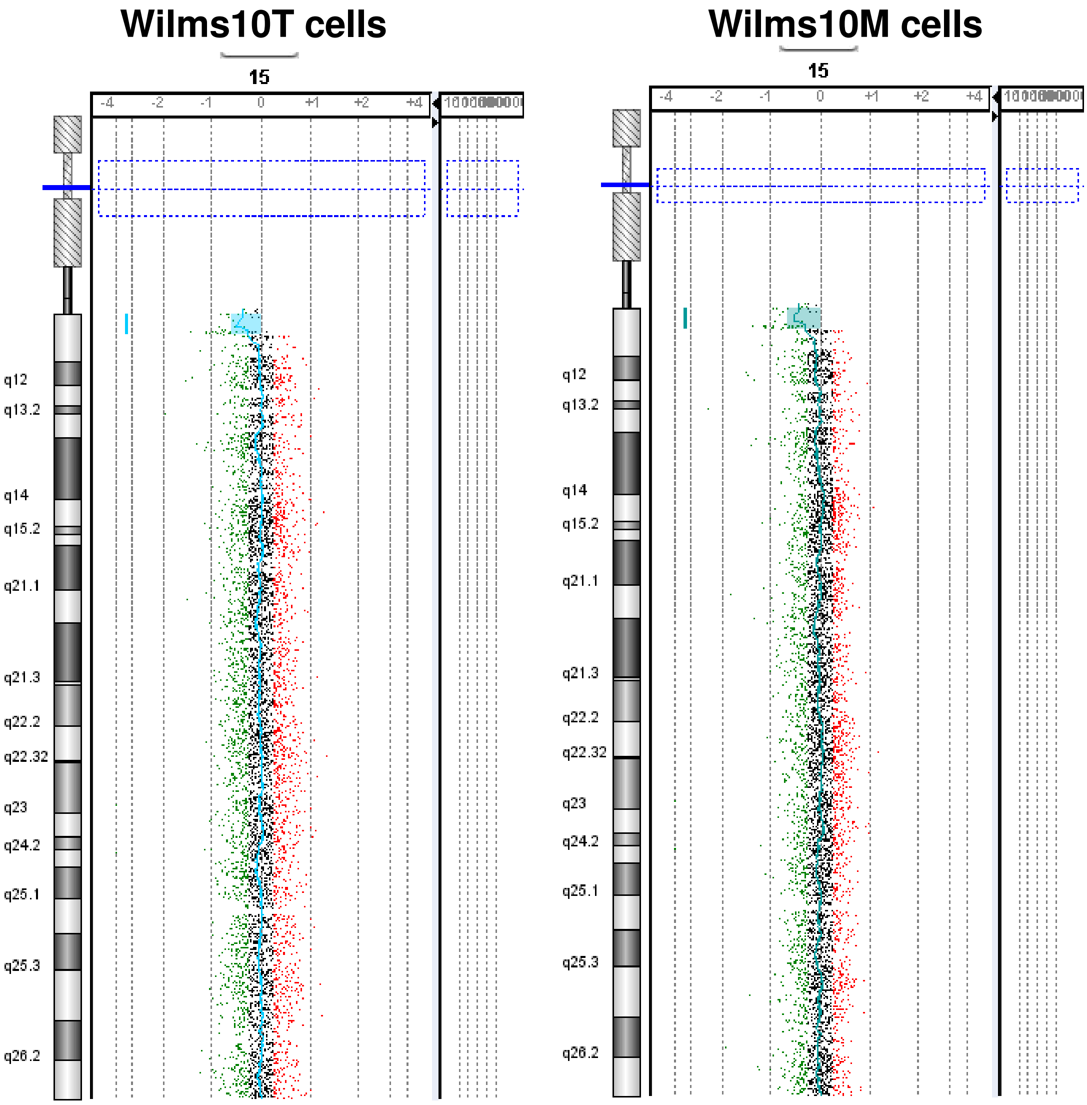

Figure 9. aCGH analysis of Wilms10T and Wilms10 M cells.
Both cell cultures have an identical germ line deletion in 15q, a known CNV

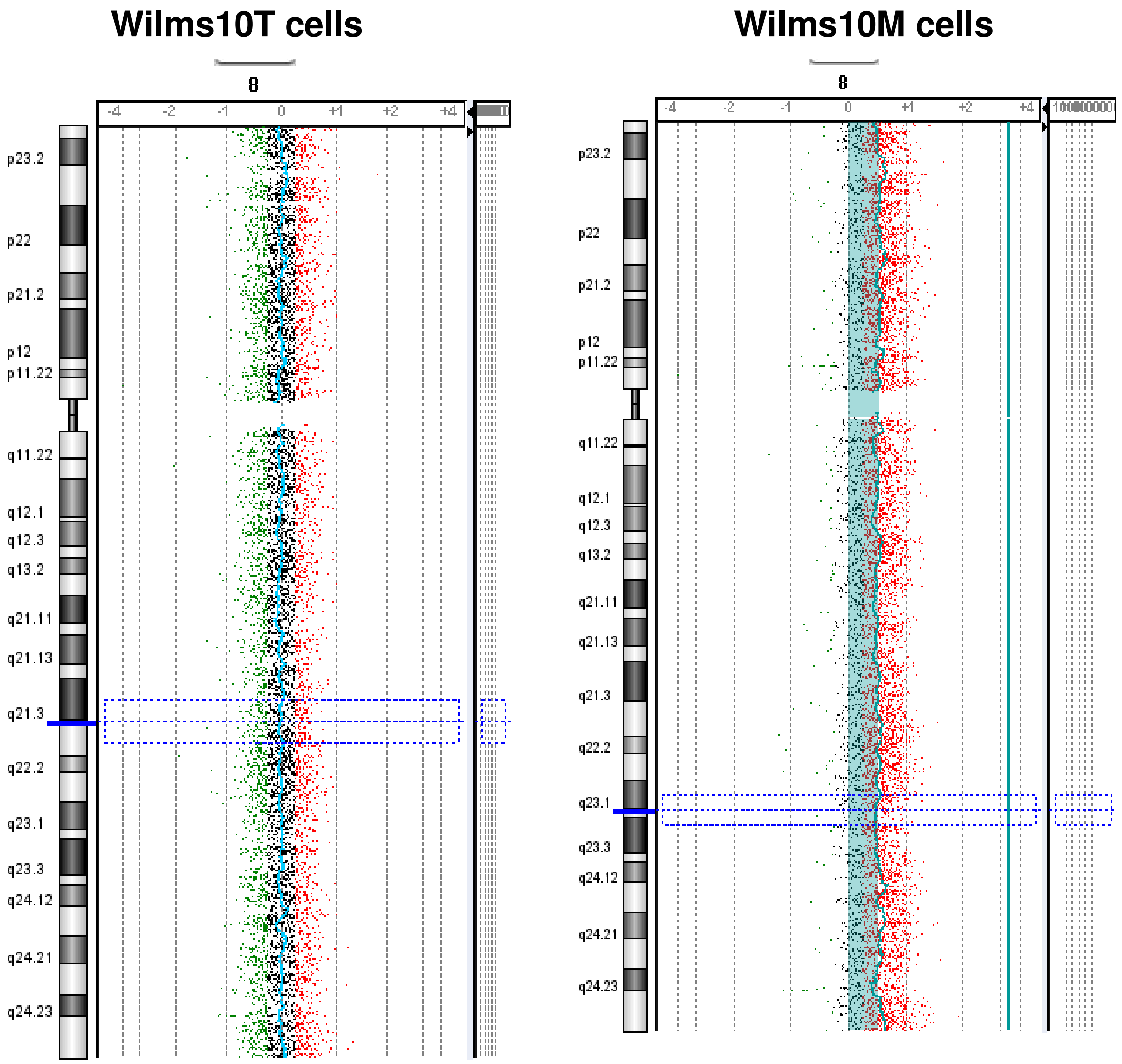

Figure 10. aCGH analysis of Wilms10T and Wilms10 M cells.
The Wilms10M cells have three copies of chromosome 11, not present in Wilms10T cells. Trisomy 8 was also verified with a chromosome 8 FISH probe in 84.4% of the cells.
