# Supplementary material for: Chemotherapy and terminal skeletal muscle differentiation in WT1‐mutant Wilms tumors
Source: Cancer Med. 2018 Mar 15;7(4):1359–68. doi: 10.1002/cam4.1379 (PMC5911586; doi:10.1002/cam4.1379)
Supplement: Supplementary file 2 — Table S1. Clinical and genetic information on tumor samples. [file CAM4-7-1359-s002.doc]

Supplementary Table S1

**Clinical and genetic** information on tumor samples

| Patient ID | WT1 mutation  cDNA | WT1 mutation  protein | Preop chemo/duration | Histology | Cells established | Gene expression tumor RNA | reference |
| --- | --- | --- | --- | --- | --- | --- | --- |
| Wilms1-1r | g: c.149C>A | p.Ser50Ter | yes 8 weeks | rhabdomyomatous | yes | yes | 1 |
| Wilms1-1l | g: c.149C>A | p.Ser50Ter | yes | rhabdomyomatous | yes | yes | 1 |
| Wilms1-2r | g: c.149C>A | p.Ser50Ter | no | triphasic | yes | yes | 1 |
| Wilms1-2l | g: c.149C>A | p.Ser50Ter | no | rhabdomyomatous | no | yes | 1 |
| Wilms2 | g:c.1084C>T | p.Arg362Ter | no | triphasic | yes | yes | 2 |
| Wilms3 | t:c.1293-1294 insA | p.Val432Serfs*87 (ext) | 4 weeks | Stromal-pred. | yes | yes | 2 |
| Wilms4 | g: del11p13;  t:c.1311-1312insC | t: p.Met437Profs*80 | no | triphasic | yes | no | 2 |
| Wilms5 | g:c.1168C>T  t: homozygous c.1297_1298delGC |  | 5 weeks | ILNR | yes | no | 2 |
| Wilms6 | g: c.1168C>T | p.Arg390Ter | 8 weeks | Stromal-pred | yes | yes | unpublished |
| Wilms8 | g: c.1168C>T | p.Arg390Ter | 8 weeks | Stromal-pred. with focal rhabdo. diff. | yes | yes | unpublished |
| Wilms10T | t:homozygous del *WT1* | p.0 | no | triphasic with focal anaplasia | yes | yes | 3 |
| Wilms10M | t:homozygous del *WT1* | p.0 | >6 months, | lung met, rhabdomyomatous | yes | yes | unpublished |
| Wilms11 | t:c.901C>T | p.Arg301Ter | no |  | yes | yes | unpublished |
| Wilms12 | g:c.1146delA | p.Phe383Serfs*16 | >8 weeks | necrotic with rhabdomyomatous differentiation, | Yes;  no growth | no | unpublished |
| Wilms13 | t:c.205_208delCACT | p.His69Profs*20 | >8 weeks | Regressive with blastema | Yes; no growth | no | unpublished |
| ANS2 | del11p13 |  | no | triphasic | nd | yes | 4 |
| WTHD1l | g:c.938C>A | p.Ser313Ter | 8 weeks | Stromal-pred | nd | yes | 5 |
| WTHD2r | g:c.368_393del29 | p.Gly123Alafs*48 | 8 weeks (including ADR) | Stromal-pred | nd | yes | 5 |
| WTHD6 | c.894+1G>C | p.Asn266Lysfs*8# | 4 weeks | Stromal-pred | nd | yes | 5 |
| WTHD9 | s:c.804insTACG | p.Glu272Val*4 | 4 weeks | Stromal-pred | nd | yes | 5 |
| WTHD11r | g: c.30_58del28 | p.Leu11Argfs*9 | 12 weeks | Stromal-pred (second tumor) | nd | yes | 5 |

l, r, left; right, Wilms1-1, first tumor left and right, Wilms1-2, second tumor left and right. g: germline mutation; t: tumor specific mutation, nomenclature for *WT1* mutations according to the transcript starting at the first in frame AUG as position1, including exon5 and KTS. For SIOP9/GPOH patients, the standard preoperative treatment was with ACTD and VCR, randomized for 4 or 8 weeks therapy

# exon6 skipping leads to two different altered RNAs depending on the presence or absence of exon5. The formula corresponds to the altered protein of long isoform containing exon5.

1. Uschkereit, C., Perez, N., de Torres, C., Küff, M., Mora, J. and Royer-Pokora, B. Different *CTNNB1* mutations as molecular genetic proof for the independent origin of four Wilms tumors in a patient with a novel germ-line *WT1* mutation. J. Med. Genet. 2007;44:393-396.
2. Royer-Pokora B, Busch M, Beier M, et al. Wilms tumor cells with WT1 mutations have characteristic features of mesenchymal stem cells and express molecular markers of paraxial mesoderm. Hum Mol Genet. 2010;9:1651–1668.
3. Brandt A, Löhers K, Beier M, et al. Establishment of a Conditionally Immortalized Wilms Tumor Cell Line with a Homozygous WT1 Deletion within a Heterozygous 11p13 Deletion and UPD Limited to 11p15. PLoS One. 2016 ;11:e0155561.
4. Drechsler M, Meijers-Heijboer, EJ, Schneider S, et al. Molecular analysis of anirdia patients for deletions involving the Wilms tumor gene. Human Genet. 1994;94:331-338
5. Royer-Pokora B, Weirich A, Schumacher V, et al. Clinical relevance of mutations in the Wilms tumor suppressor 1 gene WT1 and the cadherin-associated protein beta1 gene CTNNB1 for patients with Wilms tumors: results of long-term surveillance of 71 patients from International Society of Pediatric Oncology Study 9/Society for Pediatric Oncology. Cancer. 2008;5:1080–1089.
